# Palmitoylethanolamide Is a Disease-Modifying Agent in Peripheral Neuropathy: Pain Relief and Neuroprotection Share a PPAR-Alpha-Mediated Mechanism

**DOI:** 10.1155/2013/328797

**Published:** 2013-02-25

**Authors:** L. Di Cesare Mannelli, G. D'Agostino, A. Pacini, R. Russo, M. Zanardelli, C. Ghelardini, A. Calignano

**Affiliations:** ^1^Department of Preclinical and Clinical Pharmacology, University of Florence, Viale Pieraccini 6, 50139 Florence, Italy; ^2^Department of Experimental Pharmacology, University of Naples Federico II, 80131 Naples, Italy; ^3^Department of Anatomy, Histology and Forensic Medicine, University of Florence, 50139 Florence, Italy

## Abstract

Neuropathic syndromes which are evoked by lesions to the peripheral or central nervous system are extremely difficult to treat, and available drugs rarely joint an antihyperalgesic with a neurorestorative effect. *N*-Palmitoylethanolamine (PEA) exerts antinociceptive effects in several animal models and inhibits peripheral inflammation in rodents. Aimed to evaluate the antineuropathic properties of PEA, a damage of the sciatic nerve was induced in mice by chronic constriction injury (CCI) and a subcutaneous daily treatment with 30 mg kg^−1^ PEA was performed. On the day 14, PEA prevented pain threshold alterations. Histological studies highlighted that CCI induced oedema and an important infiltrate of CD86 positive cells in the sciatic nerve. Moreover, osmicated preparations revealed a decrease in axon diameter and myelin thickness. Repeated treatments with PEA reduced the presence of oedema and macrophage infiltrate, and a significant higher myelin sheath, axonal diameter, and a number of fibers were observable. In PPAR-**α** null mice PEA treatment failed to induce pain relief as well as to rescue the peripheral nerve from inflammation and structural derangement. These results strongly suggest that PEA, via a PPAR-**α**-mediated mechanism, can directly intervene in the nervous tissue alterations responsible for pain, starting to prevent macrophage infiltration.

## 1. Introduction

Neuropathic pain may originate from several different causes. Mechanical peripheral neuropathies are the consequence of local or extrinsic compression phenomena or impingement by an anatomic neighbor causing a localized entrapment. Traumatic neuropathies are the result of either closed injuries or open injuries to peripheral nerves [1, 2]. Both of these categories are characterized by an important inflammatory component that plays a central role in the pathogenesis of neuropathic pain. Inflammatory cells (e.g., macrophages), the production of molecules that mediate inflammation (cytokines), and the production of nervous growth factors are involved [3, 4]. In animal models it has been demonstrated that peripheral nerve injuries induce a profound local inflammatory response that involves T cells and macrophages [5]. In particular, in the neuropathic pain model induced by chronic constriction injury (CCI) an important macrophage infiltrate has been described in the damaged sciatic nerve [5–7] and in the dorsal root ganglia [8, 9]. The inflammatory response paralleled with nervous tissue alterations and pain [7, 10]. 

*N*-Palmitoylethanolamine (PEA), the endogenous amide between palmitic acid and ethanolamine, belongs to the family of fatty acid ethanolamides (FAEs), a class of lipid mediators. PEA exerts antinociceptive effects in several animal models [11, 12], prevents neurotoxicity and neurodegeneration [13, 14], and inhibits peripheral inflammation and mast cell degranulation [15]. Endogenous and exogenous PEA can modulate macrophage response [16, 17].

Anti-inflammatory effects of PEA have been associated with peroxisome proliferator-activated receptor-(PPAR-)*α* activation [18], a nuclear receptor fundamental in the control of inflammatory responses, and expressed in various cells of the immune system [19, 20]. PEA does not elicit anti-inflammatory effects in mutant PPAR-*α* null mice (PPAR-*α* ^−/−^). Indeed, when assessed in either the carrageen hindpaw or phorbol ester ear pinna tests, PEA reduced inflammation in wild-type, but not in PPAR-*α* ^−/−^, mice [18]. On the other hand, LoVerme and collegues [12] demonstrated the pivotal role of PPAR-*α* in the PEA pharmacodynamic mechanism to relieve pain.

In a mouse peripheral neuropathy model (CCI) we evaluated the effects of repeated PEA treatments on the sciatic nerve lesions responsible for neuropathic pain. Aimed to highlight the role of PPAR-*α* in PEA-evoked neurorestoration during neuropathy, a morphological study has been performed in both wild-type and PPAR-*α* null mice.

## 2. Materials and Methods

### 2.1. Animals

All procedures met the European guidelines for the care and use of laboratory animals (86/609/ECC and 2010/63/UE), and those of the Italian Ministry of Health (DL 116/92). Male wild-type (WT) and PPAR-*α* ^−/−^ (KO) (B6.129S4-SvJae-Pparatm1Gonz) mice, previously backcrossed to C57BL6 mice for 10 generations, were bred in our animal facility, where a colony was established and maintained by heterozygous crossing. Mice were genotyped as described on the supplier webpage (http://jaxmice.jax.org/), with minor modifications. DNA was extracted from tails using the RedExtract kit (Sigma-Aldrich, Milan, Italy). All animals were maintained on a 12 h light/12 h dark cycle with free access to water and standard laboratory chow.

### 2.2. Chemicals

PEA was from Tocris (Bristol, UK); it was dissolved in PEG and Tween 80 2 : 1 (Sigma-Aldrich) and kept overnight under gentle agitation with a microstirring bar. Before injection, sterile saline was added so that the final concentrations of PEG and Tween 80 were 20 and 10% v/v, respectively. Drug was injected subcutaneously (s.c.) in a dose of 30 mg kg^−1^—0.3 mL mouse—for consecutively 14 days from the day after surgery. 

### 2.3. CCI Model

The sciatic nerve of 5-6-week-old WT and KO mice were surgically ligated as described [21]. In brief, the animals were anesthetized with ketamine (100 mg kg^−1^ i.p) and xylazine (10 mg kg^−1^ i.p.), the left thigh was shaved and scrubbed with Betadine, and a small incision (2 cm in length) was made in the middle of the left thigh to expose the sciatic nerve. The nerve was loosely ligated at two distinct sites (spaced at a 2 mm interval) around the entire diameter of the nerve using silk sutures (7-0). Behavioral tests were performed on the day 14 after surgery.

### 2.4. Mechanical Hyperalgesia

We measured mechanical hyperalgesia using a Randall-Selitto analgesimeter for mouse (Ugo Basile, Varese, Italy). Latencies of paw withdrawal to a calibrated pressure were assessed on ipsilateral (ligated) paws on day 14 following ligatures. Cut-off force was set at 60 g.

### 2.5. Mechanical Allodynia

To assess for changes in sensation or in the development of mechanical allodynia, sensitivity to tactile stimulation was measured using the Dynamic Plantar Aesthesiometer (DPA, Ugo Basile, Italy). Animals were placed in a chamber with a mesh metal floor covered by a plastic dome that enabled the animal to walk freely but not to jump. The mechanical stimulus was then delivered in the mid-plantar skin of the hind paw. The cutoff was fixed at 5 g. Each paw was tested twice per session. This test did not require any special pretraining, just an acclimation period to the environment and testing procedure. Testing was performed on ipsilateral (ligated) paw on day 14 after ligation. Cutoff force was set at 5 g.

### 2.6. Tissue Explants

After the algesic test, animals were sacrificed and the ipsilateral sciatic nerves, 1 cm proximal and distal to the ligation, were explanted; the portion containing the ligature was eliminated. Contralateral nerves were also dissected, and an equivalent portion was collected. Spinal cord was removed, and lumbar section was immediately frozen in liquid nitrogen. 

### 2.7. Osmic Acid Staining

The sciatic nerve was stored in a 4% glutaraldehyde solution. The tissue samples were osmicated in 1% solution of osmium tetroxide for 2 h under constant agitation. Before and after osmication, the tissue was repeatedly rinsed in 0.1 M sodium cacodylate at pH 7.4. After gradual dehydration in ethanol, the osmicated nerve samples were embedded in paraffin (Diapath, Milan, Italy). Transverse 5 *μ*m sections were cut on a Reichert microtome (Leica, Rijswijk, The Netherlands), mounted with Canada balsam, and observed under a light microscopy.

### 2.8. Azan-Mallory Stain

After the sacrifice, the sciatic nerve was fixed in situ using 4% formalin in phosphate buffer (pH 7.4), nerves were fixed in 4% buffered neutral formalin solution, and then the nerve was embedded in paraffin. Finally 5 *μ*m sections were stained with Azan-Mallory for light microscopy studies and were graded for oedema and infiltrate [22]. The sections were semiquantified by an arbitrary scale starting from 1, mild infiltrate and oedema, up to 10, severe infiltrate and widespread oedema. The procedure was carried out by an independent researcher who was masked to the experiment.

### 2.9. Morphometry

Morphometry was performed on cross sections of osmium-fixed sciatic nerves 10 mm starting from the level of injury or at the corresponding level in uninjured control nerves. The 10 mm cross section corresponded to a level distal or proximal to the injury. Counts and measurements were carried out using ImageJ analysis software.

The first step of morphometric analysis of the sciatic nerve consisted of identifying and capturing the entire fascicle image, followed by measurement of the fascicle perimeter and area by contouring its internal epineural (magnification: objective 20x). The next step consisted of capturing sequential inner areas of the fascicle (magnification: objective 100x). The high-magnification micrographs were randomly selected in nonoverlapping areas to cover 50–75% of the total cross-sectional area of the nerve. The random selected histological images were converted into binary (black and white) images and cleaned of any blood vessels, degenerated nerve fibers, and artifacts, and the following parameters were then measured: total number and density of nerve fibers, axon diameter, and myelin sheath area. The number of small fibers, defined as fibers < 4 *μ*m, and large fibers, defined as fibers ≥ 4 *μ*m, was calculated, and the myelin thickness was determined from the difference in perimeter between the fiber and the axon. The procedure was carried out by an independent researcher who was masked to the experiment.

### 2.10. Immunohistochemistry

Sections of 5 *μ*m were deparaffinized, dehydrated, and submitted to antigen retrieval. Sections were then blocked by incubation with 3% normal goat serum for 20 min. Macrophages were detected using anti-CD86 antibody. The slides were then incubated with the primary antibodies for 18 h at 4°C. After washing in TBS, the sections were treated with the secondary antibody conjugated to Alexa Fluor 488 (1 : 1000, Invitrogen, Milan, USA) for 1 h at room temperature. Nuclei were counterstained with DAPI. Quantitative analysis of CD86 positive cells was performed collecting at least three independent fields through a 20 × 0.5 NA objective, and positive cells were counted using the “cell counter” plugin of ImageJ. 

### 2.11. Western Blot Analysis

The lumbar portion of the spinal was homogenized in lysis buffer containing 50 mM Tris-HCl pH 8.0, 150 mM NaCl, 1 mM EDTA, 0.5% Triton X-100, Complete Protease Inhibitor (Roche), and the homogenate was incubated on ice for 30 minutes. Then, the suspension was sonicated on ice using three 10-second bursts at high intensity with a 10-second cooling period between each burst. After centrifugation (13000 ×g for 15 minutes at 4°C) aliquots containing 20 *μ*g total protein underwent to western blot analysis using a mouse anti-COX2 antiserum (1 : 1000; Cell Signalling, USA). Densitometric analysis was performed using the “ImageJ” analysis software, and results were normalized to *β*-actin immunoreactivity (1 : 1000 rabbit antiserum, SantaCruz Biotechnology) as internal control. 

### 2.12. Statistical Analysis

For behavioral experiments, results were expressed as the mean ± SEM, and analyses were conducted using Statistica (Statsoft, Tulsa, OK, USA). 

Histological, morphometric, and immunohistochemical analyses were performed on 5 mice per group, evaluating 6 sciatic nerve sections for each animal. Comparison, were carried out using Mann-Whitney nonparametric tests. In all cases, the investigator was blind to the experimental status of each animal. Slides from control and experimental groups were labeled with numbers so that the person performing the image analysis was blinded as to the experimental group. In addition, all images were captured and analyzed by an investigator other than the one who performed measures to avoid possible bias. Western blot analysis were performed on 5 mice per group; comparisons were carried out using Bonferroni posttest. Data were analyzed using the ‘‘Origin 7.5” software. Differences were considered significant if *P* < 0.05 or otherwise differently reported.

## 3. Results 

**Figure 1:**
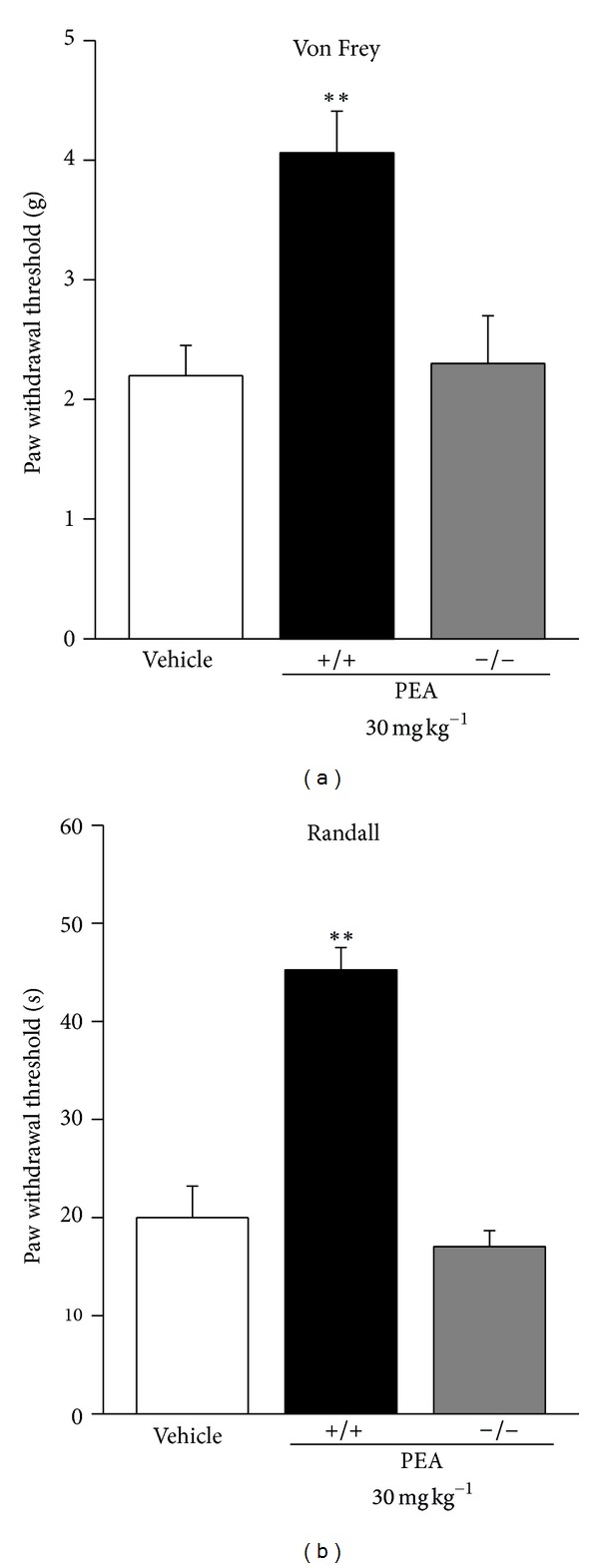
Chronic treatment effects on pain behaviour. Comparison of PEA-effects in PPAR-*α* ^+/+^ and  ^−/−^ mice. (a) Response to a mechanical noxious stimulus on the ipsilateral paw 14 days after CCI evaluated by Randall-Selitto test. (b) Response to a mechanical nonnoxious stimulus evaluated by Von Frey test. PEA (30 mg kg^−1^) was daily s.c. administered for 14 days starting from the day of operation. Each value represents the mean of 2 experiments with 12 mice per group. **P < 0.01 versus vehicle-treated mice.

PEA, (30 mg kg^−1^) s.c. daily administered starting on the day of operation, prevented pain threshold alterations elicited by CCI (Figure 1). In wild-type animals 14 days after injury, PEA reduced the hypersensitivity to a mechanical noxious stimulus (Randall-Selitto test; Figure 1(a)) as well as the hypersensitivity to a nonnoxious mechanical stimulus (Figure 1(b)). PEA efficacy against CCI-evoked pain was lacked in PPAR-*α* knock-out (−/−) mice (Figures 1(a) and 1(b)). 

**Figure 2:**
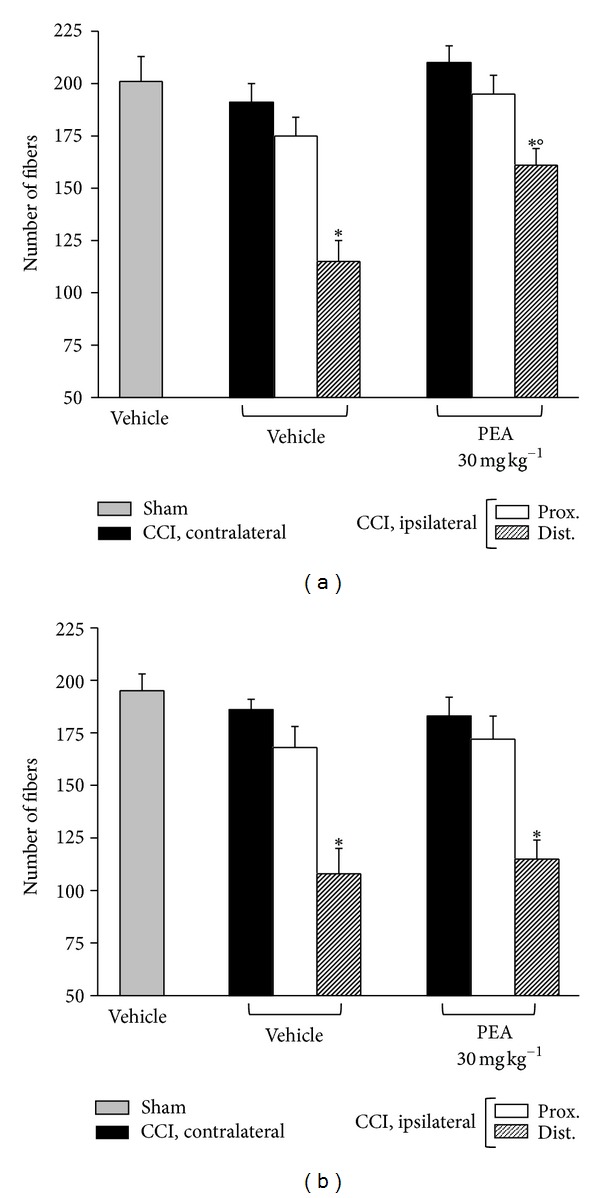
Morphometry: number of fibers. 5 *μ*m nerve sections of osmium-fixed tissues were analyzed. The distal and the proximal tract of the ipsilateral ligated nerve (CCI) was compared with the contralateral and with the sciatic nerve of sham-operated animals (sham). Effect of repeated PEA administrations (30 mg kg^−1^ s.c. for 14 days) on the number of nervous fibers in respect to vehicle-treated CCI animals and vehicle-treated sham animals. (a) PPAR-*α* ^+/+^ mice versus (b) knock-out animals. Quantitative analysis was performed evaluating 5 animals for each group. *P < 0.05 was considered as significantly different from sham, vehicle-treated mice. °*P* < 0.05 was considered as significantly different from CCI, vehicle-treated mice.

On the 14th day after injury, a morphological evaluation of the sciatic nerves was performed both on the proximal and distal parts from the ligation. 5 *μ*m sections of paraffin-embedded nerve were stained by the Azan-Mallory procedure revealing that CCI was able to induce a stronger alteration in the distal portion of the ipsilateral nerve. The histological analysis highlighted an extensive demyelination, myelin abnormalities like characteristic aggregate, generally termed “ovoids”, pathognomonic of myelin degeneration (Figure 7, arrows), and an axonal damage. As shown in Figure 2 the number of fibers was significantly reduced, particularly in the distal part of the nerve of both wild-type (+/+; graph (a)) and PPAR-*α* null (−/−; graph (b)) animals. 

**Figure 3:**
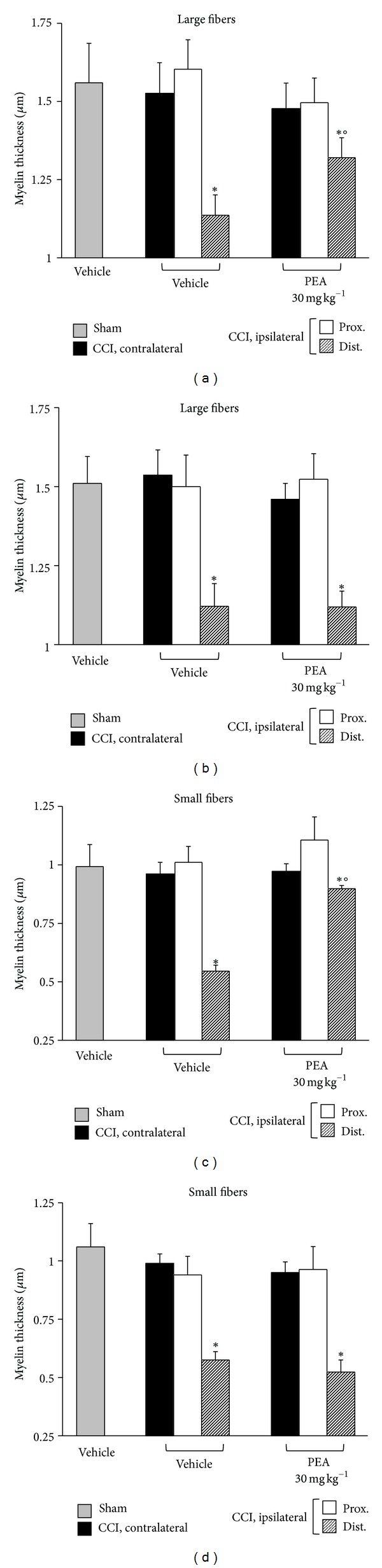
Morphometry: myelin thickness. Nerve sections (5 *μ*m) of osmium-fixed tissues were analyzed. The distal and the proximal tract of the ipsilateral ligated nerve (CCI) was compared with the contralateral and with the sciatic nerve of sham-operated animals (sham). Myelin thickness of large and small fibers of PEA-treated (30 mg kg^−1^ s.c. for 14 days) PPAR-*α* ^+/+^ ((a) and (c)) and PPAR-*α* ^−/−^ ((b) and (d)) mice in respect to saline-treated CCI and saline-treated sham animals. Quantitative analysis was performed evaluating 5 animals for each group. *P < 0.05 was considered as significantly different from sham, vehicle-treated mice. °*P* < 0.05 was considered as significantly different from CCI, vehicle-treated mice.

Morphometric evaluation of osmium-fixed tissues allowed the characterization of the alteration of the myelin sheet thickness and axonal diameter, as well as the discrimination of large against small fibers (stratified by diameter in >6 *μ*m for large and <6 *μ*m for small). CCI was able to decrease the myelin thickness of large and small fibers in the distal portion of the ipsilateral nerve in respect to the sham both in wild-type and in knock-out mice (Figures 3(a) and 3(b), large fibers; Figures 3(c) and 3(d), small fibers). 

**Figure 4:**
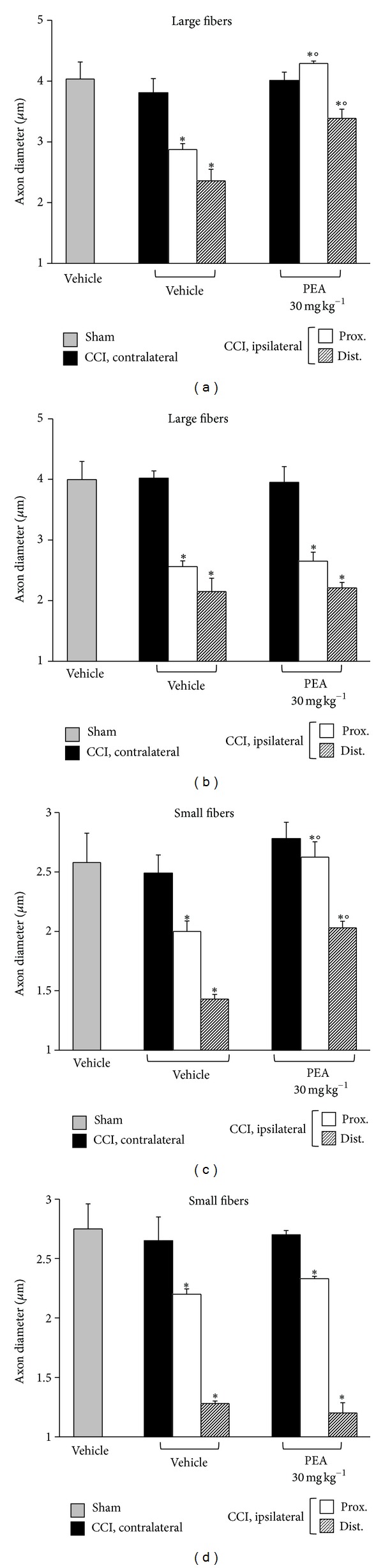
Morphometry: axon diameters. Nerve sections (5 *μ*m) of osmium-fixed tissues were analyzed. The distal and the proximal tract of the ipsilateral ligated nerve (CCI) was compared with the contralateral and with the sciatic nerve of sham-operated animals (sham). Axon diameters of large and small fibers of PEA-treated (30 mg kg^−1^ s.c. for 14 days) wild-type ((a) and (c)) and PPAR-*α* null ((b) and (d)) mice in respect to saline-treated CCI and saline-treated sham animals. Quantitative analysis was performed evaluating 5 animals for each group. *P < 0.05 was considered as significantly different from sham, vehicle-treated mice. °*P* < 0.05 was considered as significantly different from CCI, vehicle-treated mice.

In regard to axon diameter, a time-dependent decrease was revealed for all the fibers, particularly the small type, both in the distal and in the proximal portions of the ipsilateral nerve; morphometry revealed a similar profile in PPAR-*α* ^+/+^ (Figures 4(a) and 4(c)) and PPAR-*α* ^−/−^ (Figures 4(b) and 4(d)) animals. In wild-type mice repeated PEA administrations, 30 mg kg^−1^ for 14 days, were able to preserve the nerve morphology. Nerve sections of PEA-treated mice showed a higher number of fibers in respect to the saline-treated groups (Figure 2(a)); the myelin thickness in the distal portions of the nerve was decreased to a lesser extent (Figures 3(a) and 3(c)); the axon diameter was protected in the PEA group both in the proximal and in the distal nerve parts, even in the small fibers (Figures 4(a) and 4(c)). PEA was completely ineffective in PPAR-*α* null mice in preventing sciatic nerve alterations evaluated as number of fibers (Figure 2(b)), myelin thickness (Figures 3(b) and 4(d)) and axon diameters (Figures 4(b) and 4(d)).

**Figure 5:**
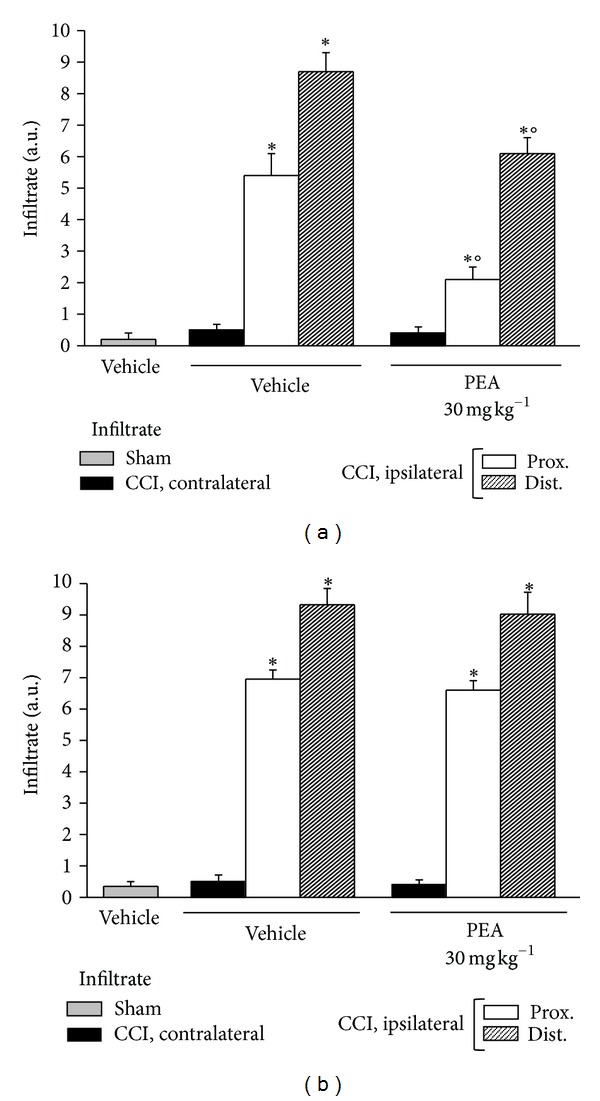
Infiltrate evaluation. On the 14th day after CCI, 5 *μ*m nerve sections of formalin-fixed tissues were analyzed by Azan-Mallory stain, and the presence of inflammatory infiltrate was evaluated and quantified by an arbitrary scale starting from 1, mild infiltrate, up to 10, severe infiltrate. Effect of PEA repeated treatments (30 mg kg^−1^ s.c. daily) was observed in (a) PPAR-*α* ^+/+^ and in (b)  ^−/−^ mice where the distal and the proximal tract of the ipsilateral ligated nerve of CCI was compared with the contralateral and with the sciatic nerve of sham-operated animals. Quantitative analysis was performed evaluating 5 animals for each group. *P < 0.05 was considered as significantly different from sham, vehicle-treated mice. °*P* < 0.05 was considered as significantly different from CCI, vehicle-treated mice.

Azan-Mallory staining revealed an abundant inflammatory infiltrate in the ligated nerve.  Figure 5 shows the infiltrate evaluation 14 days after ligation: inflammatory cells were present in the proximal and, at higher level, in the distal parts of both PPAR-*α* ^+/+^ (graph (a)) and PPAR-*α* ^−/−^ (graph (b)) mice. 30 mg kg^−1^PEA significantly prevented the cellular infiltrate number in wild-type (Figure 5(a)) but not in KO animals (Figure 5(b)). 

**Figure 6:**
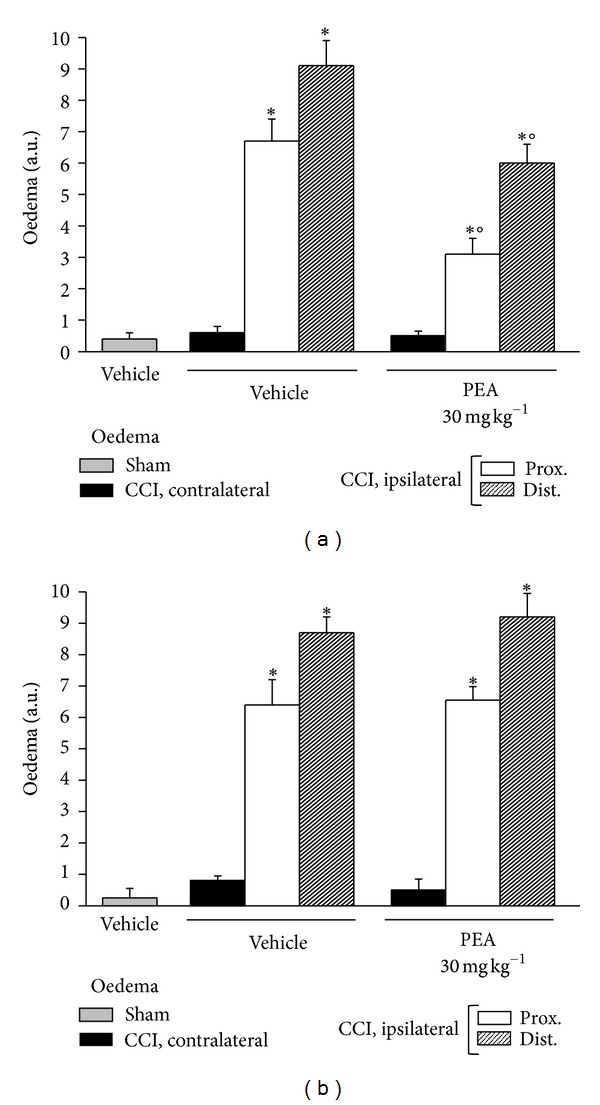
Oedema evaluation. On the 14th day after CCI, 5 *μ*m nerve sections of formalin-fixed tissues were analyzed by Azan-Mallory stain and the presence of oedema infiltrate was evaluated and quantified by an arbitrary scale starting from 1, mild oedema, up to 10, widespread oedema. Effect of PEA repeated treatments (30 mg kg^−1^ s.c. daily) was observed in (a) PPAR-*α* ^+/+^ and in (b)  ^−/−^ mice where the distal and proximal tract of the ipsilateral ligated nerve of CCI was compared with the contralateral and with the sciatic nerve of sham-operated animals. Quantitative analysis was performed evaluating 5 animals for each group. *P < 0.05 was considered as significantly different from sham, vehicle-treated mice. °*P* < 0.05 was considered as significantly different from CCI, vehicle-treated mice.

**Figure 7:**
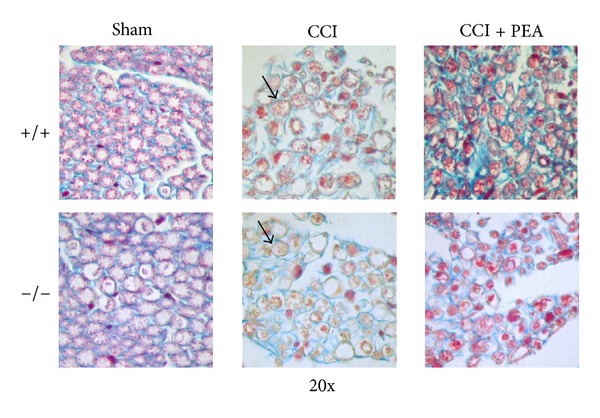
Light micrographs from 5 *μ*m transverse sections of mouse sciatic nerve stained by Azan-Mallory, comparison between PPAR-*α* ^+/+^ and PPAR-*α* ^−/−^ animals; 14th day. Sham: section of sciatic nerve from sham animals; CCI + vehicle: distal part of the sciatic nerve of injured vehicle-treated animals; CCI + PEA distal part of the sciatic nerve of CCI mice s.c. treated with 30 mg kg^−1^ PEA daily for 14 days starting from the surgery. Ovoids are evidenced by arrows. Original magnification 20x.

Moreover, both osmium fixed and Azan-Mallory-stained sections (Figure 7) allowed the observation of a massive presence of oedema among the fibers of CCI animals.  Figure 6 show the quantitative oedema evaluation 14 days after operation: the alteration was more evident in the distal portion than in the proximal one without revealable differences due to knock down PPAR-*α* gene. PEA (30 mg kg^−1^ s.c. for 14 days) was able to prevent the oedema induction of about 50% in CCI wild-type mice (Figure 6(a)). No oedema protective effects were observable in PEA-treated PPAR-*α* ^−/−^ animals in respect to vehicle (Figure 6(b)).

**Figure 8:**
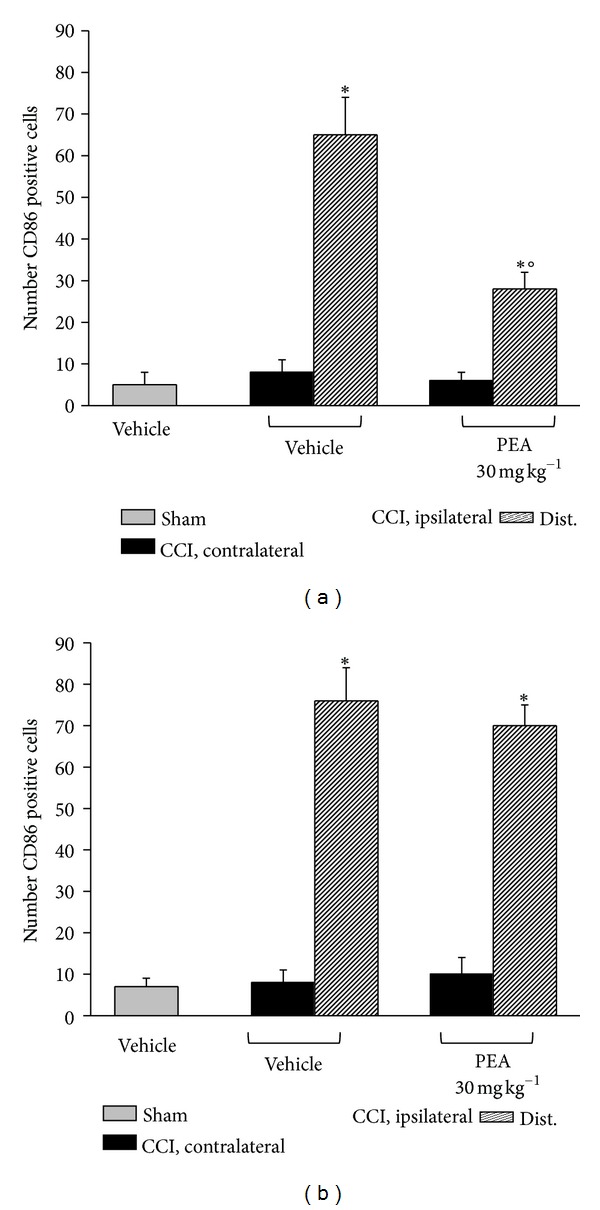
CD86 positive cells evaluation in sciatic nerve. 14 days after CCI, 5 *μ*m sections of the formalin-fixed distal part of the sciatic nerve underwent immunohistochemical staining for CD86. Effect of PEA repeated treatments (30 mg kg^−1^ s.c. daily) was evaluated in (a) PPAR-*α* ^+/+^ and in (b) PPAR-*α* ^−/−^ mice, and quantitative analysis was performed evaluating 5 animals for each group. *P < 0.05 was considered as significantly different from sham, vehicle-treated mice. °*P* < 0.05 was considered as significantly different from CCI, vehicle-treated mice.

**Figure 9:**
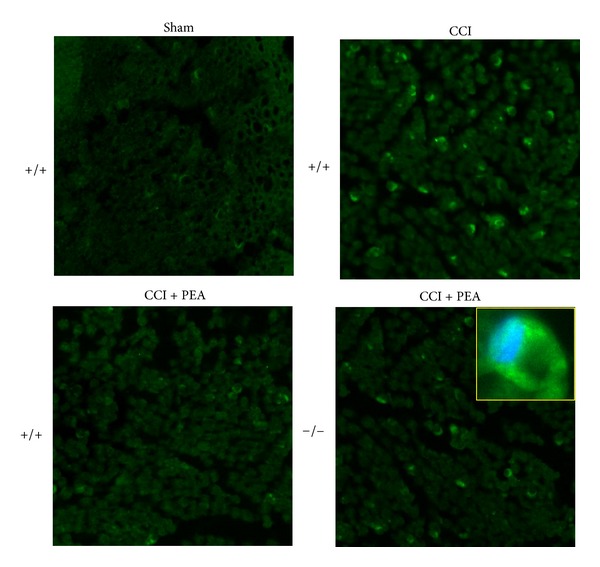
CD86 positive cells evaluation in sciatic nerve. 14 days after CCI, 5 *μ*m sections of the formalin-fixed distal part of the sciatic nerve underwent immunohistochemical staining for CD86. Effect of PEA repeated treatments (30 mg kg^−1^ s.c. daily) was evaluated in PPAR-*α* ^+/+^ and in  ^−/−^ mice, and representative images are showed, and a detailed image is shown in the insert. Original magnification 20x.

The immune inflammatory cells evaluated were diffusely distributed throughout the nerve tissue in all samples of the CCI mice, whereas a mild CD86 positive reaction was detectable in CCI mice administered with PEA as well as in sham-operated animals. PPAR-*α* ^−/−^ mice showed a persistence of macrophage infiltrate also in the nerve of PEA-treated animals (Figures 8 and 9). 

**Figure 10:**
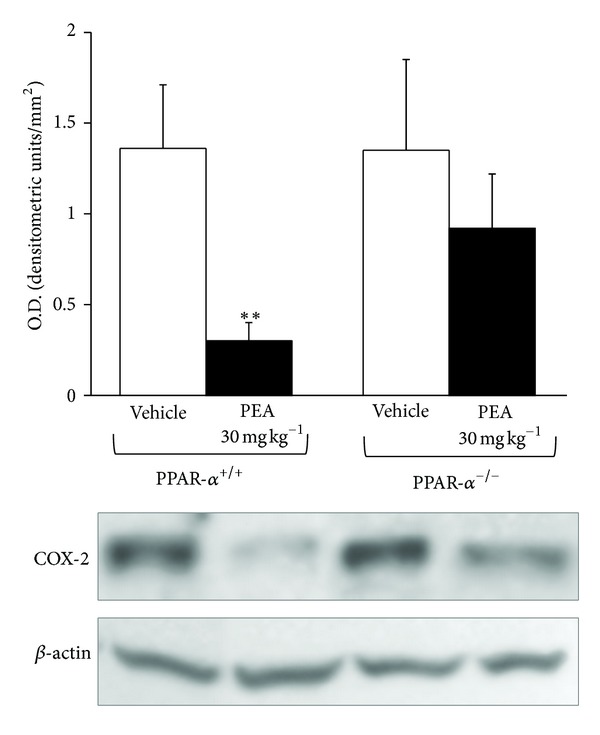
COX-2 expression levels in spinal cord. On the 14th day the spinal cord of PPAR-*α* ^+/+^ and PPAR-*α* ^−/−^ mice was analyzed by western blot. Upper panel shows the densitometric quantification of COX-2 levels in CCI vehicle-treated animal in comparison with CCI mice repetitively treated with PEA (30 mg kg^−1^ s.c. daily). Values were normalized to *β*-actin immunoreactivity. Data are expressed as the mean ± SEM of triplicate determinations performed on 5 animals for each group. **P < 0.01 was considered as significantly different from vehicle (+/+).

CCI-dependent inflammatory response in the central nervous system was evaluated measuring COX2 levels. As shown in Figure 10, in wild-type mice PEA was able to significantly prevent COX2 increase induced by nerve ligation. This anti-inflammatory effect was lost in PPAR-*α* ^−/−^ animals.

## 4. Discussion 

Pharmacological treatment of peripheral neuropathy is actually restricted to symptomatic drugs that are only partially able to control pain perception [23]. Antidepressants, antincovulsants, and opioids cannot intervene in nervous tissue alterations that act as a base of neuropathic pain. The present results describe the direct protective effect of repeated PEA treatment on lesioned peripheral nerves. 

CCI induces morphometric alterations of the sciatic nerve that dramatically affect the portion distal from the injury and that are also able to induce severe proximal impairment. At the same dose active in pain relief, PEA prevents the reduction of myelin sheet thickness and axonal diameter and improves a characteristic degeneration of myelin as highlighted by Azan-Mallory staining. According to previous results [7, 24, 25] CCI-mediated nerve architecture derangement is accompanied by a profound local inflammatory reaction which includes oedema, infiltration of hematogenous immune cells, and induction of various soluble factors like cytokines and chemokines. In particular, the present results describe a characteristic infiltrate of CD86 positive cells. CD86 is a phenotypic marker of the “classically activated” M1 macrophages stimulated by proinflammatory cytokines, as IFN*γ*, or by lipopolysaccharide and typically recruited after nervous system trauma [26]. M1 macrophages produce high levels of oxidative metabolites (e.g., nitric oxide and superoxide) and proinflammatory cytokines that are essential for host defense and tumor cell killing but that also cause collateral tissue damage [27]. The treatment with PEA attenuates the degree of peripheral inflammation, reducing oedema and macrophage infiltration allowing for hypothesizing a synergism between the anti-inflammatory and the neuroprotective mechanisms of PEA. On the other hand, an inflammation control mediated by PEA is highlighted also in the spinal cord. According to previous data CCI induces a COX-2 increase in locations of the central nervous system consistent with the neuroanatomical substrates of spinal nociception [28]. COX-2 produces prostaglandins that contribute to the development and maintenance of spinal hyperexcitability after peripheral nerve injury [28, 29]. Reducing COX-2 levels, PEA seems able to intervene also in these central mechanisms of pain chronicization. To note that a direct inhibition of pro-inflammatory cytokines, using a TNF-*α* antibody, for instance, attenuates pain-related behavior but has no effect on nerve regeneration [30].

PEA is a naturally occurring amide between palmitic acid and ethanolamine it is a lipid messenger known to mimic several endocannabinoid-driven actions even though PEA does not bind CB1, CB2, and abn-CBD receptors [31]. So far, numerous actions of PEA on immune cells such as inhibition of mast cell degranulation, attenuation of leukocyte extravasation, and modulation of cytokine release from macrophages have been described [16, 32]. Anti-inflammatory effects of PEA have been associated with peroxisome proliferator-activated receptor (PPAR)-alpha activation [18]. PPAR-*α*, well known for its role in lipid metabolism, controls transcriptional programs involved in the development of inflammation through mechanisms that include direct interactions with the proinflammatory transcription factors, NF-*κ*B and AP1, and modulation of IkB function [33]. Pharmacological studies have demonstrated that PPAR-*α* agonists are therapeutically effective in rodent models of inflammatory and autoimmune diseases [34]. Our results show that in a neuropathic pain model the PPAR-*α* genetic ablation determines a loss of PEA effectiveness in reducing oedema prevention and CD-86 positive infiltrating cells. On the other hand, the recruitment of reactive inflammatory cells and subsequent proinflammatory cascades offers a prime target for neuroprotective agents. Agonists of PPAR-*α* such as fenofibrate and Wy-14643 protect against cerebral injury by antioxidant and anti-inflammatory mechanisms and reduce the incidence of stroke in mice [35, 36]. Using a spinal cord injury model, Genovese et al. [37] demonstrated that dexamethasone utilizes PPAR-*α* to reduce inflammation and tissue injury in a rat model of spinal cord trauma. On the contrary, the PPAR-*α* agonist gemfibrozil does not promote tissue preservation and behavioral recovery after spinal contusion injury in mice [38]. Our study shows the obligatory role of PPAR-*α* for the neuroprotective effect of PEA in peripheral neuropathy. In the sciatic nerve of CCI mice PEA exerts a widespread protective effect on both myelin, and axons throughout a PPAR-*α*-mediated mechanism, since PEA treatment fails to rescue nerve tissue in PPAR-*α* knock-out animals. The neuroprotective PEA properties were suggested by Skaper et al. [39] since dose dependently PEA protected cerebellar granule cells from glutamate toxicity in neuronal single cell cultures and prevented histamine-induced cell death in hippocampal cultures. These effects were elicited without involvement of CB receptors. More recently Koch et al. [40] described the protective effect of PEA on dentate gyrus granule cells in excitotoxically lesioned organotypic hippocampal slice cultures; the specific PPAR-*α* antagonist GW6471 blocked these effects. PEA exerts neuroprotective activities in neurodegenerative diseases. In a mouse model of Alzheimer's and Parkinson's diseases, PEA reduced oxidative and apoptotic damages and improve behavioral dysfunctions by a PPAR-*α*-mediated mechanism [14, 41]. In a cellular model, PEA was able to blunt *β*-amyloid-induced astrocyte activation and, subsequently, to improve neuronal survival through selective PPAR-*α* activation [42]. 

In neuropathic conditions PPAR-*α* seems to join the antihyperalgesic, anti-inflammatory, and neuroprotective effects of PEA. On the other hand the inflammatory response to a damage is crucial for both pain sensation and tissue alterations; the importance of inflammatory mediators is well demonstrated in the pathogenesis of neuropathic pain, where infiltrating macrophages and Schwann cells may be involved in the modulation of these mediators in response to nerve injury [43].

Starting from the relevance of the PPAR-*α* in PEA antineuropathic properties, the misunderstood role of peroxisome is intriguing. Peroxisomes fulfill multiple tasks in metabolism and adapt contents and functions according to cell type, age, and organism. Among the metabolic reactions that take place in peroxisomes, oxygen metabolism, *β*-oxidation of a number of carboxylates that cannot be handled by mitochondria, *α*-oxidation of 3-methyl-branched chain and 2-hydroxy fatty acids, ether lipid synthesis, and detoxification of glyoxylate are the most important [44, 45]. Patients with peroxisomal dysfunction present with severe and diverse neurological anomalies, including neuronal migration defects, dysmyelination and inflammatory demyelination, macrophage infiltration, and axon damage, proving that these organelles are indispensible for the normal development and maintenance of the nervous system [45–47]. Thereafter, the peroxisome stimulation could be a broad spectrum approach to prevent nervous tissue damage, and a PEA, PPAR-*α*-mediated, increase in peroxisome number and/or functionality could be also hypothesized. A preclinical study [48] showed that PEA-mediated reduction of spinal cord damage was paralleled by an induction of PPAR-*α* expression and an up-regulation of potential PPAR-*α* target genes, but a clear relationship between PPAR-*α* activation and peroxisome boosting is still lacking.

## 5. Conclusions 

The present results demonstrate the neuroprotective properties of PEA in a preclinical model of neuropathic pain. Antihyperalgesic and neuroprotective properties are related to the anti-inflammatory effect of PEA and its ability to prevent macrophage infiltration in the nerve. PPAR-*α* stimulation is the common pharmacodynamic code.
