# Correction to “Palmitoylethanolamide Is a Disease‐Modifying Agent in Peripheral Neuropathy: Pain Relief and Neuroprotection Share a PPAR‐Alpha‐Mediated Mechanism”

**DOI:** 10.1155/mi/9757580

**Published:** 2026-07-08

L. Di Cesare Mannelli, G. D′Agostino, A. Pacini, et al., “Palmitoylethanolamide Is a Disease‐Modifying Agent in Peripheral Neuropathy: Pain Relief and Neuroprotection Share a PPAR‐Alpha‐Mediated Mechanism,” *Mediators of Inflammation*, 2013, 328797, https://doi.org/10.1155/2013/328797.

In the article, there are errors in Figure 9, in which duplications within and between panels have been reported directly and on PubPeer [1]. The authors explained that this was due to errors in the figure preparation and provided original images and data. After assessment by the Chief Editor, Anshu Agrawal, the results and conclusions remain unaffected. The correct Figure 9 is as follows:

**Figure 9:**
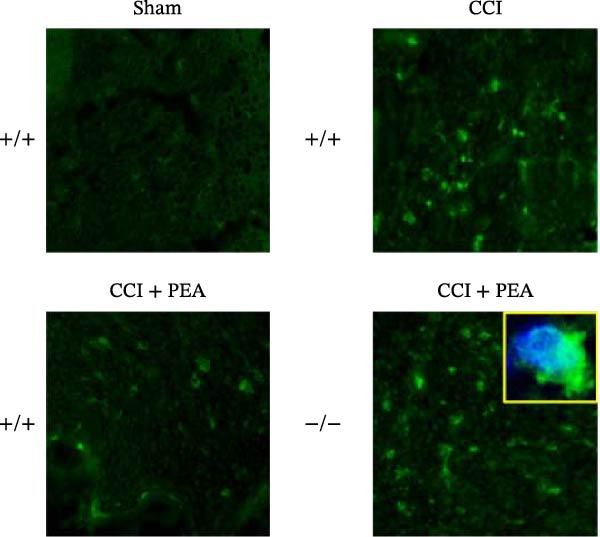
CD86 positive cells evaluation in sciatic nerve. Fourteen days after CCI, 5 μm sections of the formalin‐fixed distal part of the sciatic nerve underwent immunohistochemical staining for CD86. Effect of PEA repeated treatments (30 mg kg^−1^ s.c. daily) was evaluated in PPAR‐α^+/+^ and in ^−/−^ mice, and representative images are showed, and a detailed image is shown in the insert. Original magnification 20x.

We apologize for these errors.
